# Neuromodulation for Mild Traumatic Brain Injury Rehabilitation: A Systematic Review

**DOI:** 10.3389/fnhum.2020.598208

**Published:** 2020-12-11

**Authors:** Francesca Buhagiar, Melinda Fitzgerald, Jason Bell, Fiona Allanson, Carmela Pestell

**Affiliations:** ^1^School of Psychological Science, University of Western Australia, Perth, WA, Australia; ^2^Curtin Health Innovation Research Institute, Curtin University, Sarich Neuroscience Research Institute, Nedlands, WA, Australia; ^3^Perron Institute for Neurological and Translational Science, Sarich Neuroscience Research Institute Building, Nedlands, WA, Australia; ^4^Curtin University, Perth, WA, Australia

**Keywords:** neuromodulation, persistent post-concussive symptoms, post-concussion syndrome, mild traumatic brain injury, concussion

## Abstract

**Background:** Mild traumatic brain injury (mTBI) results from an external force to the head or body causing neurophysiological changes within the brain. The number and severity of symptoms can vary, with some individuals experiencing rapid recovery, and others having persistent symptoms for months to years, impacting their quality of life. Current rehabilitation is limited in its ability to treat persistent symptoms and novel approaches are being sought to improve outcomes following mTBI. Neuromodulation is one technique used to encourage adaptive neuroplasticity within the brain.

**Objective:** To systematically review the literature on the efficacy of neuromodulation in the mTBI population.

**Method:** A systematic review was conducted using Medline, Embase, PsycINFO, PsycARTICLES and EBM Review. Preferred Reporting Items for Systematic Reviews and the Synthesis Without Meta-analysis reporting guidelines were used and a narrative review of the selected studies was completed. Fourteen articles fulfilled the inclusion criteria which were published in English, investigating an adult sample and using a pre- and post-intervention design. Studies were excluded if they included non-mild TBI severities, pediatric or older adult populations.

**Results:** Thirteen of fourteen studies reported positive reductions in mTBI symptomatology following neuromodulation. Specifically, improvements were reported in post-concussion symptom ratings, headaches, dizziness, depression, anxiety, sleep disturbance, general disability, cognition, return to work and quality of life. Normalization of working memory activation patterns, vestibular field potentials, hemodynamics of the dorsolateral prefrontal cortex and excessive delta wave activity were also seen. The studies reviewed had several methodological limitations including small, heterogenous samples and varied intervention protocols, limiting generalisability. Further research is required to understand the context in which neuromodulation may be beneficial.

**Conclusions:** While these positive effects are observed, limitations included unequal representation of neuromodulation modalities in the literature, and lack of literature describing the efficacy of neuromodulation on the development or duration of persistent mTBI symptoms. Better clarity regarding neuromodulation efficacy could have a significant impact on mTBI patients, researchers, clinicians, and policy makers, facilitating a more productive post-mTBI population. Despite the limitations, the literature indicates that neuromodulation warrants further investigation. PROSPERO registration number: CRD42020161279.

## Introduction

An estimated 99 in 100,000 people of all ages experience a traumatic brain injury (TBI) in Australia, with numbers varying between states (Pozzato et al., 2019). Estimates from a study in New Zealand are much higher with 790 in 100,000 experiencing TBI (Feigin et al., 2013). Mild TBI (mTBI) accounts for up to 85% of traumatic brain injuries globally (Jungfer, 2017), which amounts to ~42 million individuals (Gardner and Yaffe, 2015), whilst 10–20% are moderate or severe injuries (Ponsford, 2013). Some common causes of mTBI include cycling accidents, motor vehicle accidents, falls, assaults and sports injuries (Jagnoor and Cameron, 2014; Langer et al., 2020). A comprehensive review of the literature is provided in this introduction to give context for the systematically reviewed material.

### Terminology

The terms concussion and mTBI are often used interchangeably, especially in the context of sports-related injuries (McCrory et al., 2013), however they do not always refer to the same thing. The most recent consensus statement for sports-related concussion states that concussion is a subset of less severe mTBI, where altered brain function results from a traumatic injury (Harmon et al., 2019). Outside the sporting context, the definition put forward by the American Congress of Rehabilitation Medicine states that mTBI is “a traumatically induced physiological disruption of brain function, as manifested by at least one of the following: (1) any period of loss of consciousness (2) any loss of memory for events immediately before or after the accident (3) any alteration in mental state at the time of the accident (e.g., feeling dazed, disoriented, or confused) and (4) focal neurological deficit(s) that may or may not be transient but where the severity of the injury does not exceed the following: loss of consciousness of ~30 min or less after 30 min an initial Glasgow Coma Scale (GCS) of 13–15 and posttraumatic amnesia (PTA) not >24 h” (American Congress of Rehabilitation Medicine, 1993). The more recent International Classification of Disease (ICD-11), simply defines concussion as a loss of consciousness caused by injury (World Health Organization, 2018).

It has been recommended that a more unified nomenclature would enhance consistency in the literature, with some authors suggesting that mTBI is a more appropriate and specific term (Sharp and Jenkins, 2015). The term mTBI will be used in this article to encompass both concussion and mTBI. Further disparity regarding terminology exists in relation to the term post-concussion syndrome (PCS), with some using it to refer to the immediate sequalae of concussion, whilst others refer to the prolonged presence of symptoms (Rose et al., 2015). Additionally, while the International Collaboration on Mild Traumatic Brain Injury (Donovan et al., 2014) suggests using the term post-traumatic symptoms, since the symptoms are not specific to concussion, the more recent position statement of the American Medical Society for Sports Medicine (Harmon et al., 2019), recommends using the term persistent post-concussive symptoms (PPCS). Since there is no general consensus on the use of these terms, and the term PPCS is used in recent literature (McInnes et al., 2017; Mercier et al., 2020), the latter will be used here to refer to a delayed or abnormal response to mTBI.

### Mild TBI Symptoms

The symptoms resulting from mTBI can be divided into six categories cognitive, vestibular, ocular, headache/migraines, anxiety/mood and fatigue (Harmon et al., 2019). Affective symptoms may include irritability, depression, anxiety and emotional lability (Katz et al., 2015; Bergersen et al., 2017). Somatic symptoms often include dizziness, sleep disturbance, headaches, nausea, photophobia, visual disturbance, and phonophobia. Commonly reported cognitive symptoms include difficulties with memory and attention, decreased processing speed, higher distractibility, multitasking difficulties, foggy feeling, and difficulty maintaining a train of thought (Katz et al., 2015; Bergersen et al., 2017). Another commonly reported symptom following mTBI is fatigue, which is considered a complex symptom having multiple exacerbating and contributing factors including cognitive exertion, sleep disturbance, chronic stress, somatic symptoms and mental health (Cicerone and Kalmar, 1994; Ouellet and Morin, 2006; de Leon et al., 2009; Bay and de-Leon, 2011). Whilst there is a wide variation of symptom patterns following mTBI (Faul et al., 2010), multiple mTBIs are more likely to lead to worse outcomes compared to a single mTBI (Belanger et al., 2010; Mez et al., 2017).

### Persistent Symptoms

There is a lack of consensus about the expected duration of symptoms following mTBI, with expert physicians giving timelines ranging from 2 weeks to 3 months; whereas patients can report much longer symptom durations (Sharp and Jenkins, 2015). Stemming from this, the point at which the symptoms become persistent is also a matter of debate, where some consider symptoms remaining beyond the expected 2-week recovery timeframe to be persistent (Harmon et al., 2019), while others consider 1 month (McCrory et al., 2017) and 3 months (American Psychiatric Association, 2000) to be the transition point to persistent symptoms. On average the symptoms of a single mTBI last 2 weeks in adults, however 10–20% of individuals go on to develop PPCS (Carroll et al., 2014; Rivara and Graham, 2014). In fact, up to 40% of people experiencing mTBI will endure PPCS for longer than 3 months (Cnossen et al., 2018), and of those with persistent symptoms, 80% will still experience impairments 12 months after injury (McMahon et al., 2014). This lingering disorder is characterized by physical, cognitive and emotional regulation deficits which can persist for months to years including dizziness, headaches, insomnia, fatigue, mood swings, and cognitive impairment (Willer and Leddy, 2006; Carroll et al., 2014; Rivara and Graham, 2014; Sharp and Jenkins, 2015; Grandhi et al., 2017). Whilst cognitive function is often regained swiftly following mTBI, a recent review reported that out of 45 studies, ~50% of adults with mTBI had persistent cognitive dysfunction 3 months after injury (McInnes et al., 2017). The presence of PPCS may limit the return to pre-injury activities of daily living, such as work or sport (Lingsma et al., 2015) and can occur following even the mildest TBI.

The search for predictors of outcomes following mTBI has included various structural, neuropsychological and physiological markers, with most investigations being limited to a single measure and producing mixed findings (Bazarian et al., 1999; Sheedy et al., 2006; Topolovec-Vranic et al., 2011; Allanson et al., 2017). Risk factors for the development of PPCS have been identified in two categories, those that occur before injury and those that occur after. Longer recovery has been associated with having lower education levels, being older in age (Lingsma et al., 2015), having pre-existing psychiatric conditions (Carroll et al., 2004; Donnell et al., 2012), personality traits such as neuroticism (Merritt et al., 2015; Beidler et al., 2017), learning difficulties (Zemek et al., 2013) migraine headaches (Jotwani and Harmon, 2010; Origenes et al., 2019) and being female (Ponsford et al., 2012). Specifically, females tend to have a higher number of symptoms at the time of injury and higher rates of persistent symptoms (Guinto and Guinto-Nishimura, 2014). Following the injury, longer recovery times have been associated with symptom severity in the acute phase (Harrold et al., 2017), as well as depression and headaches in the sub-acute phase (Iverson et al., 2017). Hence, mTBI represents a significant problem which has implications for the individual and society in general.

### Rehabilitation: Current Trends

Given the above statistics on symptom recovery following mTBI, for the purposes of this review one could consider two broad timepoints for treatment one is from the time of the event up to 2 weeks post injury (early intervention), and the other is after 2 weeks (post-acute intervention). Up until recently, mTBI was not thought to require much clinical intervention beyond observation (Mann et al., 2017). However, emerging evidence about the potential for chronic functional impairment after mTBI (Dikmen et al., 2010; McMahon et al., 2014; de Koning et al., 2017; Theadom et al., 2017), has resulted in a more proactive early intervention approach (Collins et al., 2016; Leddy et al., 2016; McCrory et al., 2017; Harmon et al., 2019).

Following a mTBI, the general consensus guidelines put forward by the American Medical Society for Sports Medicine (Harmon et al., 2019), suggest that the first priority is to rule out any cervical spine injuries or neurological emergencies and to consider whether brain imaging is indicated (Silverberg et al., 2019). Once the individual is medically stable, education is provided about the nature of mTBI, the expectation of a short recovery process and how to manage any symptoms that may arise (Government of South Australia, 2009; Prince and Bruhns, 2017). A recent review on the evaluation and treatment of mTBI suggests that education limits the development of persistent symptoms and a lack of education and patient discharge information seems to be associated with complex recovery, reducing the likelihood that an individual will follow up with a neurologist in the presence of persistent symptoms (Prince and Bruhns, 2017). Finally, when the symptoms are stable a gradual return to daily activities, without symptom exacerbation is encouraged (Harmon et al., 2019; Silverberg et al., 2019). Although this outlines the recommended approach, a systematic and comprehensive approach may not always be used.

In fact, as reported by Prince and Bruhns (2017), there is a paucity of research addressing treatment of PPCS in mTBI, with methodological inconsistencies being a major factor identified in the literature. This gap in the evidence base for mTBI treatment, warrants further exploration by researchers and clinicians alike, of potential interventions that may assist individuals with persistent symptoms in the post-acute phase (Silverberg et al., 2019). This may include vestibular and ocular assessments, reviewing medication side-effects, biochemical investigations, screening for depression and anxiety, as well as cognitive neuropsychological assessments where indicated (Silverberg et al., 2019). Overall, the management of PPCS tends to be symptom focussed and includes medication, behavioral and physical therapy as well as lifestyle changes (Stilling et al., 2019b). Despite the comprehensive approach to the clinical management of mTBI, between 10 and 40% of people report persistent symptoms (Carroll et al., 2014; Rivara and Graham, 2014; Cnossen et al., 2018; Stilling et al., 2019b). It has been suggested that the reasons for limited treatment success to date, is the fact that the interventions do not address the pathophysiological cascade responsible for the persisting symptoms (Hadanny and Efrati, 2016). Indeed, it is likely that we do not yet have a comprehensive understanding of the pathophysiology underlying mTBI and PPCS (Huang et al., 2017). Other factors that influence recovery include individual factors such as pre- and post-injury characteristics as well as injury characteristics (Polinder et al., 2018), and motivations for secondary gain (Patrick and Horner, 2014). Consequently, research has focussed on further understanding the pathophysiological sequalae of mTBI as well as novel interventions to modulate this process, in order to reduce the functional impact associated with persistent symptoms following mTBI (Girgis et al., 2016; Hadanny and Efrati, 2016; Leung et al., 2016a; Stilling et al., 2019b).

### Neuromodulation

One example of novel approaches to the management of mTBI is non-invasive neuromodulation. This approach represents the interface between technology and the nervous system, through the process of modification, stimulation, inhibition, regulation or activity altering input into the autonomic, peripheral or central nervous system (Krames et al., 2009). The proposed mechanism for positive outcomes is mediated through adaptive neuroplasticity. The neuromodulation techniques are thought to restore altered function within the brain which results in better functioning and reduced symptomatology. This study sought to review the effects of certain types of neuromodulation on the symptoms of mTBI and PPCS. Whilst there is emerging evidence for the use of these techniques in traumatic brain injury, to our knowledge there is no systematic review of the literature which is specific to mTBI in adults. The types of neuromodulation we intended to review included repetitive transcranial magnetic stimulation (rTMS), transcranial direct current stimulation (tDCS), transcutaneous vagus nerve stimulation (tVNS), neurofeedback (NF) and Photobiomodulation (PBM), including low level laser therapy (LLLT). These approaches will be discussed in turn below by considering their mechanism of action, broader clinical applications, and potential side effects. Since no studies utilizing PBM or tVNS, were included in our review, we did not include them below. The following section will provide background information about the neuromodulation techniques covered in the review, the use of these modalities in mTBI will be covered in the body of the systematic review.

### Repetitive Transcranial Magnetic Stimulation (rTMS)

This therapeutic modality creates a magnetic field via an electromagnetic coil which is placed near the scalp, inducing cortical excitation both under the site of stimulation and at distant areas via synaptic connections (Chen et al., 1997; Ziemann et al., 1998). Cortical excitation is induced by high frequency stimulation (5 Hertz or above), while lower frequency stimulation (1 Hertz) reduces cortical excitation (Peinemann, 2004; Mansur et al., 2005). Its use in the mTBI population is of interest, as research has found rTMS to be beneficial for other brain based disorders such as various types of chronic pain (Leung et al., 2009; de Andrade et al., 2011; Lee et al., 2012; Misra et al., 2013) and post-stroke aphasia (Allen et al., 2012). In fact, rTMS is approved by the Food and Drug Administration (FDA) as a treatment for major depression in the United States of America (Brunelin et al., 2007). Whilst it is typically well-tolerated, common side effects of rTMS include temporary headache, localized pain, paraesthesia, and toothache (Rossi et al., 2009). On rare occasions, it has also been known to cause seizures (Rossi et al., 2009; Lefaucheur et al., 2014).

### Transcranial Direct Current Stimulation (tDCS)

Whilst rTMS is considered a neurostimulator due to its capacity to induce action potentials via rapid membrane depolarisation without contacting the scalp, tDCS is a neuromodulator involving direct contact with the scalp (Nitsche et al., 2008). tDCS has the capacity to change spontaneous excitation of the brain by altering the membrane's resting potential (Wagner et al., 2007; Nitsche et al., 2008), via a one to two milliamp current which flows between two rubber electrodes placed on the scalp (Nitsche et al., 2008; Villamar et al., 2012). The anode increases cortical stimulation whilst the cathode lowers it (Nitsche and Paulus, 2000; Nitsche et al., 2003). To date, the research literature has reported that tDCS may have a positive impact on various psychiatric conditions including depression, substance addictions (Kekic et al., 2016), post-traumatic stress disorder, generalized anxiety disorder (Shiozawa et al., 2014) and obsessive compulsive disorder (OCD; Palm et al., 2017). Overall, tDCS is considered a safe technique (Herrera-Melendez, 2019), however some mild side effects have been reported including skin irritation, tingling discomfort, headache, burning sensation at site of application and fatigue (Brunoni et al., 2011).

### EEG Neurofeedback

Biofeedback, a form of operant conditioning using physiological measures, can take many forms and neurofeedback refers to the category of biofeedback which involves measures of brain function (May et al., 2013). In the current review we focused on a sub-category of neurofeedback which utilizes electroencephalography (EEG) as a measure, sometimes referred to as EEG neurofeedback. EEG involves recording the brain's electrical activity through scalp surface electrodes (Kane et al., 2017). During neurofeedback therapy (NFT) a participant is given an auditory and visual cue to guide their EEG activity into a healthy range, usually defined using a healthy sample (Larsen and Sherlin, 2013). Whilst the participant is not doing this by subjectively altering their thoughts (Othmer et al., 2005), they are required to understand the concept and attend to the task (May et al., 2013). NFT has been reported to improve executive and cognitive functions, memory, attention, motor recovery and seizures following mild, moderate and severe TBI (Tinius and Tinius, 2000; Walker et al., 2002; Duff, 2004; Thornton and Carmody, 2005; Tan et al., 2009), migraine (Stokes and Lappin, 2010), depression (Choi et al., 2011; Linden et al., 2012), anxiety (Hammond, 2005), OCD (Surmeli and Ertem, 2011; Koprivova et al., 2013), and schizophrenia (Surmeli et al., 2012). NFT has also been shown to enhance fractional anisotropy, gray and white matter volume in moderate TBI (Munivenkatappa et al., 2014) and normal participants (Ghaziri et al., 2013). Moreover, improvements in quality of life, cognition and magnetic resonance imaging abnormalities were observed in a TBI sample (Reddy et al., 2014). Side effects from NFT tend to be transient, they may be due to the treatment procedure or the chosen stimulation protocol and may include headaches, mood swings, nightmares, nausea and tiredness (Rogel et al., 2015).

The following sections will describe the methods and findings of the systematically reviewed studies reporting the effects of neuromodulation on the symptoms of mTBI and PPCS. Specifically, rTMS, tDCS, and neurofeedback will be covered.

## Methods

### Search Strategy

**Figure 1:**
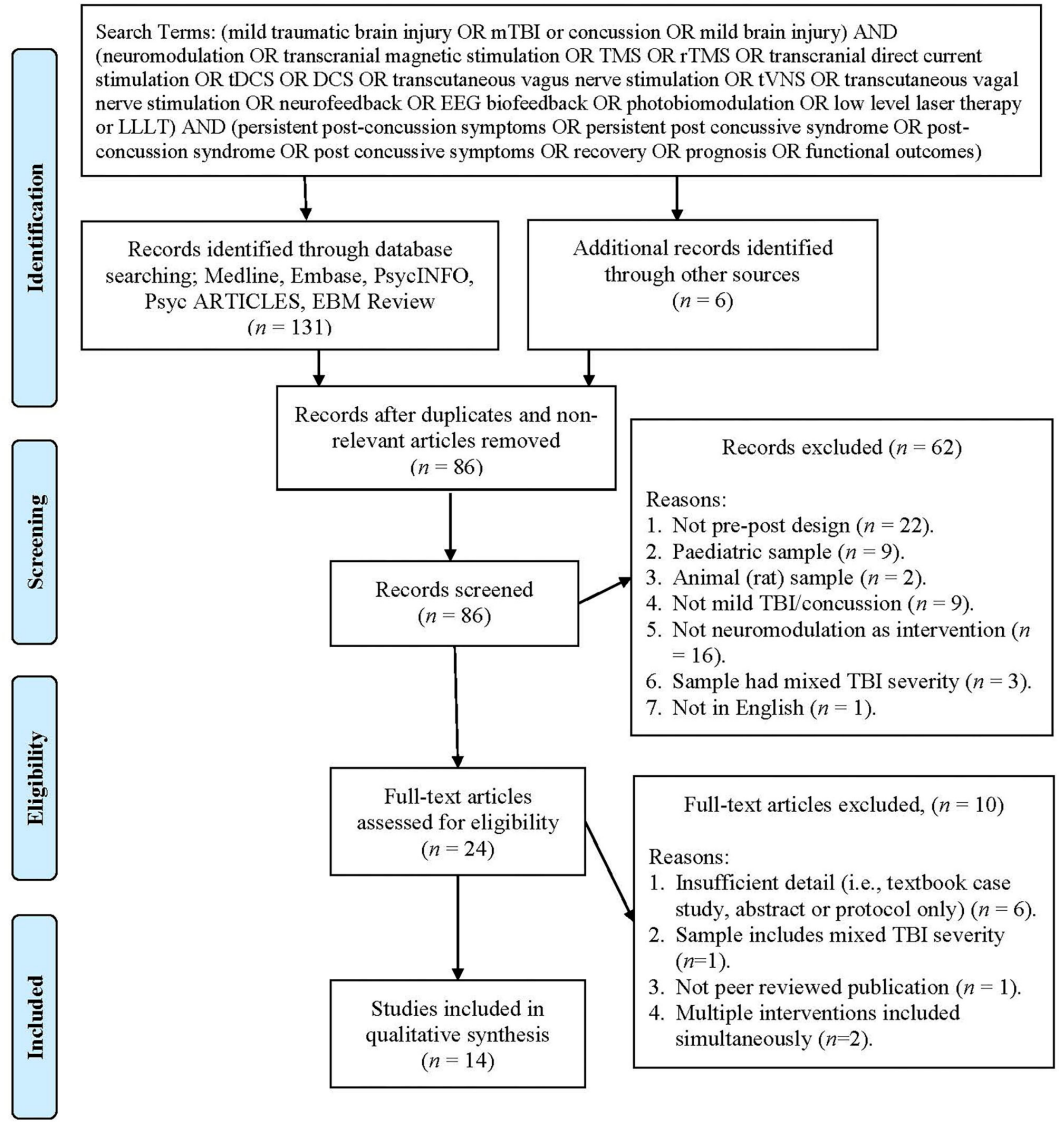
PRISMA flow chart showing the process of study selection (Moher et al., 2009).

A systematic search of the literature using five databases was performed (Medline, Embase, PsycINFO, PsycARTICLES, and EBM Review) including all studies up until the seventh of December 2019, with no limitations. The search terms used included (mild traumatic brain injury or mTBI or concussion or mild brain injury) and (neuromodulation or transcranial magnetic stimulation or TMS or rTMS or transcranial direct current stimulation or tDCS or DCS or transcutaneous vagus nerve stimulation or tVNS or transcutaneous vagus nerve stimulation or neurofeedback or EEG biofeedback or photobiomodulation or low level laser therapy or LLLT) and (persistent post-concussion symptoms or persistent post concussive syndrome or post-concussion syndrome or post concussive symptoms or recovery or prognosis or functional outcomes). References listed in eligible studies were also examined to identify any studies missed by electronic searching. Procedural and reporting methods were based on the preferred reporting items for systematic reviews (PRISMA) guidelines (see Figure 1 in Moher et al., 2009) as well as the synthesis without meta-analysis (SWiM) reporting guidelines (Campbell et al., 2020) where possible. From the initial studies listed (*n* = 131) and hand searched articles (*n* = 6), duplicates and irrelevant articles were removed (*n* = 51) and 86 studies were assessed using the inclusion criteria, leading to a further 62 articles being excluded. Full texts of the remaining 24 articles were reviewed, and a further 10 articles were excluded (see Figure 1 for exclusion reasons), resulting in 14 articles being included in this review. Data items included population: adults who experienced a mTBI or concussion, intervention: types of neuromodulation including tDCS, rTMS, neurofeedback, LLLT/photobiomodulation, tVNS, outcomes: levels of persistent post-concussion symptoms.

### Study Selection Criteria

The protocol for this study was registered on the International Prospective Register of Systematic Reviews (PROSPERO; registration number: CRD42020161279) and can be accessed at (https://www.crd.york.ac.uk/prospero/display_record.php?RecordID=161279). The studies evaluated in this review fulfilled the following inclusion criteria published in English, investigating an adult sample and using a pre- and post-intervention design. The exclusion criteria consisted of: studies with non-mild TBI severities (e.g., moderate/severe TBI or acquired brain injury) due to the varying nature and underlying pathophysiology of these injuries. Studies investigating pediatric or older adult populations (<18 and >65 years old) were also excluded due to the neurodevelopmental/neurodegenerative changes seen in these populations which may impact their results.

### Screening, Data Extraction and Management

Study selection and data extraction was conducted by the first author (FB) and independently reviewed by another author (FA), concordance was 100%. Information extracted from the studies included: study setting study population, participant demographics, baseline characteristics, intervention and control conditions, study methodology, completion rates, outcomes and suggested mechanisms of action of the interventions. Although the pre-registered study protocol included ascertaining whether neuromodulation reduced delayed recovery from mTBI, no information was found relating to this question in the included studies, so it was not addressed in this review. Additionally, none of the studies screened that utilized PBM or tVNS met the inclusion criteria, so these modulation techniques were also excluded from the review. The narrative results were grouped by type of intervention since they vary in their mechanism of action as well as the number of brain areas targeted. An attempt was made to classify the results tables using the RoB tool (Sterne et al., 2019), however due to the lack of standardized procedures for reaching an overall judgement in observational studies, this was not used. Instead studies within the tables were grouped as randomized and non-randomized, and then listed in alphabetical order for simplicity. For the most part, this also adhered to the order of highest to lowest quality studies based on the level of evidence assessment.

### Assessment of Methodological Quality

Assessment of methodological quality was conducted using the Cochrane handbook of Systematic Reviews of Interventions (Higgins et al., 2019), a risk of bias tool (Sterne et al., 2019) and a published guide for assessing risk of bias (RoB) in observational studies (Viswanathan et al., 2013). RoB was assessed in five domains for randomized trials including bias due to, randomization, deviations from intended intervention, missing data, outcome measurement and selection of reported result. Studies were judged as low, moderate or high RoB based on the algorithms provided in the RoB tool version 2 (Sterne et al., 2019). Observational studies were judged as low, moderate or high based on authors' judgements (FB and CP), as no specific guide for reaching an overall judgement was provided. We also used The Oxford 2011 Levels of Evidence table to assess the quality of evidence (OCEBM Levels of Evidence Working Group, 2011) based on study design, across five levels of evidence (1 = highest, 5 = lowest). The level of evidence and RoB analysis was conducted by two authors (FB and CP) and any discrepancies were resolved by discussion.

We did not perform a meta-analysis of the results due to the heterogeneity of the included study designs, the variety of interventions and outcome measures used as well as the lack of availability of detailed data within the studies. A narrative synthesis was conducted because there was a mix of randomized controlled trials and non-randomized designs and the studies did not contain sufficient data to use any of the recommended data synthesis methods provided in the Cochrane handbook (Higgins et al., 2019). Where possible the difference between the pre- and post-intervention means was used, however this was not always available, despite attempting to contact study authors for further detail. In such cases a significance value was used where available.

## Results

The 14 studies selected for this review included case reports (*n* = 4), pilot experimental studies (*n* = 3), uncontrolled open trials (*n* = 1) and randomized controlled trials (*n* = 6). Supplementary Table 1 summarizes the characteristics of the selected studies including study design, population, age and gender of participants, intervention used, whether they had a control group, initial sample size, follow up time after the last intervention session, completion rate, whether blinding was present and the sources of funding or conflicts of interest listed. The mean number of participants across all studies was 17.7 (range = 1 to 44), with a mean age of 40.4 years (range = 18 to 69) and 62.7% being male. Follow up times ranged from zero to 6 months, with an average completion rate of 84%. The neuromodulation intervention techniques utilized in the studies included rTMS (*n* = 11), neurofeedback (*n* = 2), and tDCS (*n* = 1).

### Risk of Bias and Quality of Evidence

Supplementary Table 2 provides a summary of the RoB assessments for the randomized studies. All six studies were assessed as having a low RoB. Supplementary Table 3 summarizes the non-randomized studies RoB assessment. All eight studies were assessed as having a high risk of bias, on most occasions this was due to limitations inherent in the study design. The included studies ranged in level of evidence from level 2 (*n* = 6) to level 4 (*n* = 1) and 5 (*n* = 7), as summarized in Supplementary Tables 2, 3.

Supplementary Table 4 summarizes the injury characteristics of participants across all included studies. In reviewing the terminology and diagnostic criteria used within these studies, the terms concussion and mTBI were mostly used interchangeably when referring to the head injury. Injury classifications included mTBI (*n* = 6), sports-related concussion (*n* = 1), mTBI with PCS (*n* = 1), PPCS (*n* = 1), PPCS with post-traumatic headache (*n* = 1), mTBI-related headache (*n* = 1), mTBI with post traumatic headache (*n* = 2) and mild closed head injury (*n* = 1). The terminology used for persistent symptoms included post-concussion syndrome (*n* = 1), post-concussive symptoms (*n* = 1), PPCS *n* = 2, persistent post-traumatic symptoms (*n* = 1) and symptom-specific terminology such as persistent post traumatic headache (*n* = 1), mTBI-related headache (*n* = 3), post traumatic dizziness of idiopathic origin (*n* = 1), depression (*n* = 1) and chronic pain (*n* = 1).

The persistent symptom-duration also varied between studies, with some considering a minimum 6-month symptom duration as inclusion criteria (*n* = 4), whilst others selected 3 months (*n* = 4), 1 month (*n* = 1), or an unlimited timeframe (*n* = 5). Not all the included studies listed the diagnostic criteria used and some of those listed included more than one diagnostic criteria. They included: Post-Concussion Symptom Scale (Lovell et al., 2006) score of 21 or more (*n* = 2), GCS of 13–15, PTA ≤ 24 h and LOC for <30 min (*n* = 1), Veterans Affairs and Department of Defense diagnostic criteria (*n* = 3), International Classification of Headache Disorder (ICHD-2; *n* = 2), ICHD-3 (*n* = 1), International Classification of Diseases (*n* = 1), American Congress of Rehabilitation Medicine (*n* = 2), Consensus Statement on Concussion in Sport−5th International Conference (*n* = 2), World Health Organization (*n* = 1) and the American Academy of Neurology Practice (*n* = 1). Despite the various labels and diagnostic criteria, a common factor was that a GCS of 13–15, a PTA of 24 h or less and a LOC <30 min defined an mTBI, which is the same classification used earlier in the terminology section of this review.

Of the 14 studies included, four reported no findings on brain imaging and 10 did not report on imaging. Details about loss of consciousness (LOC), post-traumatic amnesia (PTA) and Glasgow Coma Scale (GCS) score were not reported in 8, 9 and 10 studies, respectively. In the studies reporting these details, LOC ranged from nil to <1 h (*n* = 1), PTA ranged from nil to <24 h, and the GCS score ranged from 13 to 15. Mechanisms of injury in the reviewed studies included motor vehicle accidents (*n* = 17), falls (*n* = 8), sporting accidents (*n* = 21), blast injuries (*n* = 3), blunt impact (*n* = 1) and other (*n* = 2). Fifty percentage of the studies did not report mechanism of injury or did not specify the proportion of each mechanism within the sample (*n* = 1). Time since injury ranged from 3 months to 28 years.

The information regarding pre-injury characteristics of the participants in the included studies was limited. Information about intoxication at the time of injury was not reported by any of the 14 studies. The number of previous head injuries was reported in 57% of the studies and ranged from zero to seven, with an average of 1.93 head injuries per participant. Pre-existing conditions amongst the participants included depression (*n* = 9 participants), drug addiction (*n* = 1 participant), migraine (*n* = 1 participant), and other medical conditions (*n* = 4 participants). The participants' post-injury characteristics are summarized in Supplementary Table 5. The duration of symptoms for the participants ranged from 3 months to 28 years, with large within-sample variations for most studies. The symptom domains recorded included cognitive dysfunction (*n* = 5 studies), fatigue (*n* = 3 studies), sleep disturbance (*n* = 2 studies), mood dysregulation (*n* = 7 studies), headaches or migraines (*n* = 5 studies), ocular dysfunction (*n* = 2 studies), vestibular dysfunction (*n* = 3 studies), physical symptoms (*n* = 5 studies), and other (*n* = 3). Participants sought a variety of interventions prior to engaging with the included studies such as medication, physical therapies such as massage, physiotherapy, exercise, craniosacral therapy, vestibular rehabilitation, prism glasses and vision therapy, botox, psychology, social work, and productivity consultation.

Supplementary Table 6A summarizes the interventions used in the included studies. Among the 11 rTMS studies, eight applied stimulation to the left dorsolateral prefrontal cortex (DLPFC), two to the motor cortex and one to both the DLPFC and the motor cortex. The number of sessions varied from three to 20, with an average of 11.3 sessions. The study using tDCS applied stimulation over the motor cortex for 1 session. Of the two neurofeedback studies, one applied feedback at two scalp electrode sites and another at five scalp electrode sites. Fifty percentage of the studies had a control group which received sham stimulation. The measures used comprised several imaging techniques including MRI, MRI angiogram, functional MRI (fMRI), diffusion MRI (dMRI), diffusion tensor tractography (DTT), diffusion tensor imaging (DTI), magnetic resonance spectroscopy (MRS), magnetoencephalography (MEG), quantitative electroencephalography (qEEG), electrovestibulography (EvestG), videonystagmography (VNG), TMS and functional near infrared spectroscopy (fNIRS). Additionally, a multitude of neuropsychological assessment measures were used including measures of cognition, information processing and mental state, post-concussion symptoms, headache quality, depression and anxiety, post-traumatic stress disorder and quality of life.

Across the 14 studies, several neurophysiological changes were observed following mTBI, including microstructural damage in the corpus callosum (Ansado et al., 2019), reduced haemodynamic activation in the DLPFC (Stilling et al., 2019a), altered working memory network activation patterns (Koski et al., 2015; Ansado et al., 2019) and excess delta frequency EEG (Huang et al., 2017). The integrity of the spinothalamocortical pathway was also altered in participants with mTBI and chronic pain (Choi et al., 2018). Thirteen studies reported positive outcomes, with one study stating that their outcomes did not reach statistical significance or meet the minimal clinically important difference criteria and one study reported a negative outcome. Since a standardized vote counting method was not used, the reader is advised to consider the RoB and level of evidence assessments (Supplementary Tables 2, 3) when reviewing the specific outcomes detailed in Supplementary Table 6B.

Specifically, rTMS was effective for improving activation in several areas within the working memory network (Koski et al., 2015; Ansado et al., 2019), increasing information processing speed and verbal fluency (Fitzgerald et al., 2011), reducing overall post-concussion symptom ratings (Koski et al., 2015; Moussavi et al., 2019; Stilling et al., 2019a), chronic pain levels (Choi et al., 2018), headache intensity, frequency and duration (Koski et al., 2015; Leung et al., 2016a,b, 2018; Stilling et al., 2019a), dizziness (Paxman et al., 2019), depression ratings (Fitzgerald et al., 2011; Leung et al., 2018; Moussavi et al., 2019), anxiety (Stilling et al., 2019a), general disability measures (Fitzgerald et al., 2011), and improving quality of life (Paxman et al., 2019; Stilling et al., 2019a). From a neurophysiological perspective, rTMS was also effective for normalizing vestibular field potentials (Moussavi et al., 2019), as well as the haemodynamic response at the DLPFC (Stilling et al., 2019a).

Neurofeedback was effective for reducing excessive delta wave EEG activity (Huang et al., 2017) as well as post-concussion symptom scores (Walker et al., 2002; Huang et al., 2017) and sleep disturbance (Huang et al., 2017). Improved rates of return to work were also seen with both rTMS and neurofeedback (Walker et al., 2002; Stilling et al., 2019b). Anodal tDCS did not influence gamma-aminobutyric acid (GABA) concentration or receptor activity in the primary motor cortex (Wilke et al., 2017).

Whilst these techniques are generally safe with seven studies reporting no side effects, some adverse events have been reported in the included studies. In the rTMS studies adverse events included symptom aggravation (Koski et al., 2015; Ansado et al., 2019; Moussavi et al., 2019; Stilling et al., 2019b), headaches (Koski et al., 2015; Moussavi et al., 2019), toothache (Stilling et al., 2019b), vertigo, sleep disturbance (Koski et al., 2015), stimulation site sensitivity (Koski et al., 2015; Leung et al., 2016b; Stilling et al., 2019b), dizziness (Leung et al., 2016b; Stilling et al., 2019b), and fatigue (Paxman et al., 2018). No side effects were reported in the neurofeedback studies.

## Discussion

This study has systematically reviewed the literature on the efficacy of neuromodulation as a rehabilitation tool for the mTBI population, up until December 2019. Since this is an emerging area of research, limited studies met the inclusion criteria for review. Additionally, of the 14 studies included, only six were rated as having a low risk of bias (Leung et al., 2016b, 2018; Wilke et al., 2017; Choi et al., 2018; Moussavi et al., 2019; Stilling et al., 2019b), and the other eight studies had several methodological limitations (Walker et al., 2002; Fitzgerald et al., 2011; Koski et al., 2015; Leung et al., 2016a; Huang et al., 2017; Paxman et al., 2018; Ansado et al., 2019; Stilling et al., 2019a). At face value, all but one of the studies demonstrated that neuromodulation had a positive effect on the various symptoms measured and sometimes neurophysiological functioning following mTBI, as detailed in Supplementary Table 6B. The study that found no effect was not measuring symptoms, but rather GABA receptor concentration/ activation (Wilke et al., 2017).

From a symptom perspective, neuromodulation was reported to be effective at improving post-concussion symptom ratings (Walker et al., 2002; Koski et al., 2015; Huang et al., 2017; Moussavi et al., 2019; Stilling et al., 2019a,b), pain and headaches (Leung et al., 2016a,b, 2018; Choi et al., 2018; Stilling et al., 2019a,b), dizziness (Paxman et al., 2018), depression (Leung et al., 2018; Moussavi et al., 2019; Stilling et al., 2019a,b), anxiety (Stilling et al., 2018), sleep disturbance (Huang et al., 2017), general disability (Fitzgerald et al., 2011), some aspects of cognition (Fitzgerald et al., 2011), return to work (Walker et al., 2002; Stilling et al., 2019a,b), and quality of life (Choi et al., 2018; Paxman et al., 2018; Stilling et al., 2019a,b). From a neurophysiological perspective, efficacy was seen in normalizing altered working memory activation patterns (Koski et al., 2015; Ansado et al., 2019), vestibular field potentials (Moussavi et al., 2019), haemodynamic responses within the DLPFC (Stilling et al., 2019a) and excessive delta wave EEG activity (Huang et al., 2017). However, we recommend caution in interpreting these findings due to the several methodological limitations within most of the included studies. The studies using neurofeedback and tDCS did not report any side effects or adverse events, while seven of the 11 studies using rTMS reported side effects.

### Strengths and Limitations of the Reviewed Studies

Completion rates were high throughout most of the studies, with an average of 84%. One might argue that findings in such motivated participants may not be generalisable to the general population. Declared conflicts of interest were minimal with only two studies involving authors who had an interest in the neuromodulation technology being used. Appropriate outcome measures were utilized in many of the studies, including standardized measures and neuroimaging techniques. Self-report measures were also used due to the nature of symptoms being measured and these were not considered inappropriate in most cases. However, caution must be taken when a self-report measure is used in a non-blinded participant, which was the case for several included studies, further increasing the potential for bias. The current literature demonstrates that despite the positive outcomes, many participants were not symptom free following intervention, indicating that further research is needed. When reviewing the strengths of the study designs for the above findings, only six of the studies used a randomized controlled design, and whilst six studies had a sample size between 20 and 44 participants, eight studies had 15 or less participants. Hence, the generalisability of the results is limited when combining the relatively small sample sizes with the lack of a control group for most of the studies.

Whilst these outcomes are promising, generalisability is limited due to the wide range of methodological procedures employed. Besides the considerations of risk of bias, the intervention protocols used varied between studies, particularly the number of intervention sessions undertaken by the participant. This highlights that the field of neuromodulation is still emerging, and standardized protocols are not yet available. Contributing to this uncertainty, is the lack of comprehensive understanding regarding the pathophysiology of mTBI as well as the neurophysiological mechanisms of action for each of the neuromodulation techniques. In fact, not all studies included pre and post intervention neurophysiological measures, limiting our ability to understand the full effect of neuromodulation, which in its nature, influences the brain's neurophysiology.

### Neurophysiological Mechanisms

Given the heterogeneity of the mTBI population and the myriad of potential tracts, networks and brain areas that may be altered following injury, it may be naïve to think that “one size” may in fact fit all. Perhaps the lack of efficacy of current treatment regimens for persistent symptoms following mTBI, is in part due to their symptom-focused nature rather than focusing on restoring neurophysiological function. Individualized protocols based on the individual's altered neurophysiology may be required for full rehabilitation, further emphasizing the need to incorporate functional neuroimaging and detailed neurophysiological assessment for mTBI sufferers as standard practice. The proposed mechanism of action for neuromodulation therapies is to restore altered function within the nervous system, resulting in better functioning and reduced symptomatology (Krames et al., 2009). So, an alternative approach to rehabilitation using neuromodulation, might be to focus on normalizing neurophysiological aberrations rather than symptoms. Of course, symptom reduction would still be the main goal, however it would be anticipated that improved neurophysiological function would be correlated with reduced symptomology. In this way, neuromodulation opens a doorway for effecting change within the neurophysiological system, a phenomenon that has not been readily available thus far.

Moreover, it is yet to be seen whether certain types of neuromodulation might be more beneficial at specific time points in the recovery period following mTBI. Only one of the 14 studies divided their sample into short- and long-term PCS (Moussavi et al., 2019), and significant findings were only seen in the short-term PCS group. This raises concern about the strength of findings in samples with a large range of symptom duration/time since injury, since one sub-group might be skewing the data favorably for an otherwise non-responsive sub-group. Additionally, efficacy may be impacted by the type of neuromodulation used, in that the mechanism of action for one type of neuromodulation may be more beneficial at certain stages of the recovery period. Although there is some theoretical understanding of the mechanism by which each modality effects change, a detailed understanding of which neurophysiological phenomena are correlated with the worst functional outcomes for mTBI sufferers at each stage of the recovery period is still emerging. This reinforces the need for neurophysiological analysis both post injury as well as pre- and post-intervention.

Finally, the literature is still heterogenous in its use of key terms relating to mTBI, concussion, PCS and PPCS. Whilst the criteria for diagnosing mTBI were relatively homogenous, there is still debate about whether concussion and mTBI are in fact interchangeable, in which case the terms post-concussion syndrome and persistent post-concussion symptoms may not be appropriate to use for post-mTBI sequalae.

This study was also limited in that the systematic search did not equally represent all the types of neuromodulation that we set out to cover. Specifically, no studies utilizing PBM or tVNS met the inclusion criteria, only two studies using neurofeedback were included and a single study using tDCS. Six studies were excluded due to having a mixed severity sample or multiple simultaneous interventions. Additionally, none of the included studies measured the efficacy of neuromodulation on the development or duration of persistent symptoms following mTBI. The field of research exploring the efficacy of neuromodulation for mTBI is heterogenous. Not all modalities are equally comprehensive in their ability to modulate the nervous system, and the training required to operate and interpret the various intervention modalities varies significantly across different types of neuromodulation. Inherently, these differences may contribute to the uneven representation of the various modalities in the scientific literature. It is recommended that future research combines both symptom-based outcome measures and neurophysiological measures to enable a better understanding of the neurophysiological effects of neuromodulation in the mTBI population, as well as the correlation between those neurophysiological effects and the presenting symptoms. Using both types of measures will also facilitate a better understanding of the potential underlying neurophysiological differences between treatment responders and non-responders. While utilizing standardized protocols for neuromodulation may detract from the unique feature of this modality to individualize interventions, it would be valuable to ensure that all parameters are reported in standardized units and nomenclature. Additionally, it is recommended that future research stratifies the sample by taking confounding variables into account. While inclusion criteria will depend on the population being sampled, the statistical analysis should factor in variables such as symptom duration/ time since injury, severity of symptoms, mechanism of injury, the age of the individual and the number of previous head injuries. Finally, we echo previous recommendations that a unified and specific nomenclature for mTBI is adopted in future research; we prefer mTBI and PPCS.

In conclusion, the heterogeneity in both the clinical features of mTBI populations and in the techniques that are termed “neuromodulation” preclude making any systematic conclusions. It is not yet possible to state with certainty that the neuromodulation techniques reviewed are effective for reducing post-mTBI symptoms, however initial findings are encouraging and further research using more robust methodological designs, is required to determine the context in which these may be appropriate rehabilitation tools. While the above recommendations may improve research methodology, the field of neuromodulation in the mTBI population highlights the challenges of individualized intervention, which may not be amenable to the standardized protocol approach used in current health care practices. Whilst treating a well-defined disorder may be straightforward, functional disorders require a much more complex approach to treatment. Improved clarity on the efficacy of neuromodulation has the potential to significantly impact the life of individuals with mTBI, by facilitating better understanding for researchers, clinicians, and policy makers alike leading to better functional outcomes for individuals following mTBI. Despite its limitations, this literature indicates that further investigation into neuromodulation for mTBI is warranted.

## Data Availability Statement

The raw data supporting the conclusions of this article will be made available by the authors, without undue reservation.

## Author Contributions

FB: conception and design of project, conducted search, study selection and data extraction, data analysis, interpretation and synthesis, risk of bias assessment and manuscript writing. MF: conception and design of project, data interpretation, manuscript critical review. JB: conception and design of project, data interpretation, manuscript critical review. FA: reviewed study selection and data extraction. CP: conception and design of project, data interpretation, risk of bias assessment, manuscript critical review. All authors: contributed to the article and approved the submitted version.

## Conflict of Interest

The authors declare that the research was conducted in the absence of any commercial or financial relationships that could be construed as a potential conflict of interest.
