# Supplementary material for: Neuromodulation for Mild Traumatic Brain Injury Rehabilitation: A Systematic Review
Source: Front Hum Neurosci. 2020 Dec 11;14:598208. doi: 10.3389/fnhum.2020.598208 (PMC7759622; doi:10.3389/fnhum.2020.598208)
Supplement: Supplementary file 1 [file Table_1.docx]

Table 1

*Summary of Included Studies Evaluating the Efficacy of Neuromodulation in mTBI*

| *Source* | *Study design* | *Population* | *Age & gender of participants (years)* | *Intervention* | *Control group* | *Initial sample* | *Follow up time* | *Completion rate* | *Blinding* | *Funding/ conflict of interest* |
| --- | --- | --- | --- | --- | --- | --- | --- | --- | --- | --- |
| *Randomised studies* | | | | | | | | | | |
| G. S. Choi et al. (2018) | Prospective randomized controlled clinical trial (pilot) | Chronic central pain following mTBI, sensory deficit in unilateral body, minimum NRS pain score 4/10, lasting at least 6 months.  Hospital setting | Males (*n*=6), females (*n*=6).  Age *M*: 42.6 years (*SD*: 8.7), range 30-56 | rTMS | Yes | 12 (controls *n*=6),  (+ *n*=30 controls for DTT imaging) | 1 month | 100% | Yes (participants) | No conflicts declared |
| Leung, Shukla, et al. (2016) | Randomised controlled experimental design | Veterans with mTBI-HA.  Outpatient setting | Males (*n*=21), females (*n*=3).  Treatment group (*n*=12) age *M*: 41.2 years, *SD*:14, Control group (*n*=12) *M*: 41.4 years, *SD*: 11.6 | rTMS | Yes | 29 | 1 month | 82.8% | Yes (participants) | Funding: VARRDA |
| Leung et al. (2018) | Prospective randomised sham-controlled study | Veterans with mTBI-HA  (NRS 3+/10, M-VAS 30/100) | Males (*n*=23), females (*n*=6).  Age *M*: 34.2 years (*SD*: 7.9) | rTMS | Yes | 44 | 1 month | 65.9% | Yes (participants) | Funding: VARRDA & the DOD Congressionally Directed Medical Research Program Grant |
| Moussavi et al. (2019) | Randomised placebo-controlled and double-blind clinical trial | PPCS, Hospital setting | Males (*n*=9), females (*n*=9).  Age *M*: 49.5 years, *SD*: 12.4 | rTMS | Yes | 22 | 2 months | 81.8% | Yes | MITACS and Manitoba Public Insurance. One author has <0.5% in the supporting company Neural Diagnostics Pty. Ltd. |
| Stilling, Paxman, et al. (2019) | Pilot double blind randomised controlled trial | PPCS + PTH, Outpatient setting | Male (*n*=2), female (*n*=18).  Age *M*: 36 years, *SD*: 11.4, range: 18-65 | rTMS | Yes | 20 | 6 months | 100% | Yes | Funding: University of Calgary, Cumming School of Medicine, Clinical Research Fund Grant, Hotchkiss Brain Institute and Department of Clinical Neurosciences Pilot Fund Grant |
| Wilke et al. (2017) | Randomised controlled experimental design | Recurrent mTBI (minimum 2 episodes).  Outpatient & hospital setting | Male (*n*=15), female (*n*=2).  Age *M*: 24.3 years, *SD*: 2.8  Controls *M*: 26.1, *SD*: 5.4 | atDCS | Yes | 39 (controls *n*=22) | Nil | NR | No | Funding: Deutsche Forschungsgemeinschaft, Bundesministerium fur Bildung und Forschung.  One author is part of the clinical scientist program funded by the Charite Universita tsmedizin Berlin and the Berlin Institute of Health. |
| *Non-randomised studies* | | | | | | | | | | |
| Ansado et al. (2019) | Pilot experimental | PPCS >6 months, PCSS score 21+ Outpatient clinic | Males (*n*=5), females (*n*=3).  Age *M*: 30 years (*SD*: 9), range 21-45 | rTMS | No | 15 | Nil | 80%  (*n*=12 completed, *n*=8 analysed) | No | Funding: CIHR |
| Fitzgerald et al. (2011) | Case report | History of severe, recurrent depression following mTBI | 41-year-old female | rTMS | No | 1 | Nil | 100% | No | None reported |
| Huang et al. (2017) | Pilot experimental study | Chronic mTBI | Males (*n*=5), females (*n*=1).  Age: 27-41 years | Neurofeedback | No | 6 | Nil | 83.3% | No | Funding: US Department of Veteran Affairs, 2 authors were associated with IASIS Technologies Inc., and Mind Brain Training Institute, however their involvement was restricted to offering training and technological advice. |
| Koski et al. (2015) | Pilot experimental study | mTBI and PCS >3 months, PCSS score >21.  Outpatient setting. | Males (*n*=9), females (*n*=6).  Age: *M*: 34.3 years, *SD*: 10.8, range: 20-60 | rTMS | No | 15 | 3 months | 80% | No | Funding: CIHR.  1 author holds several paid advisory board positions. |
| Leung, Fallah, et al. (2016) | Prospective case series | mTBI and constant headaches (NRS rating 4+/10) | Males (*n*=6).  Age: *M*:50.2 years, *SD*: 4.4, range: 38-60 | rTMS | No | 6 | Nil | 100% (one participant had 2-year gap) | No | None reported |
| Paxman et al. (2018) | Single case report | mTBI | 61-year-old male | rTMS | No | 1 | 3 months | 100% | No | None reported |
| Stilling, Duszynski, et al. (2019) | Two-patient case study | PPCS, Outpatient setting | Males (*n*=2), 47 and 49 years old | rTMS | No | 2 | 1 month | 100% | No | Funding: CIHR, Natural Sciences and Engineering Research Council of Canada |
| Walker et al. (2002) | Uncontrolled open trial | PPCS for 3+months | Males (*n*=12), females (*n*=14).  Age *M*: 39 years, range: 25-65 | Neurofeedback | No | 36 | Nil | 72.2% | No | None reported |

*Note:* atDCS = anodal transcranial direct current stimulation, CIHR = Canadian Institutes of Health Research, CST = compensatory strategy training, DOD = Department of Defense, *M* = mean, MHI = mild head injury, mTBI = mild traumatic brain injury, mTBI-HA = mTBI-related headache, M-VAS = mechanical visual analogue scale, NRS = numerical rating scale, PCS = post-concussion syndrome, PCSS = Post-Concussion Symptom Scale (Lovell et al., 2006), PPCS = persistent post-concussion symptoms, PTH = post-traumatic headache, rTMS = repetitive transcranial magnetic stimulation, *SD* = standard deviation, TBI = traumatic brain injury, VARRDA = Veteran Affairs Rehabilitation and Research Development Award.
