# Supplementary material for: Neuromodulation for Mild Traumatic Brain Injury Rehabilitation: A Systematic Review
Source: Front Hum Neurosci. 2020 Dec 11;14:598208. doi: 10.3389/fnhum.2020.598208 (PMC7759622; doi:10.3389/fnhum.2020.598208)
Supplement: Supplementary file 2 [file Table_2.docx]

Table 2. Risk of bias and level of evidence associated with included randomised studies

| Study Authors | D1 | D2 | D3 | D4 | D5 | Overall Bias | Level of Evidence |
| --- | --- | --- | --- | --- | --- | --- | --- |
| **Choi, Kwak, Lee, and Chang (2018)** | Low | Low | Low | Low | Low | Low | 2 |
| **Leung, Shukla, et al. (2016)** | Low | Low | Low | Low | Low | Low | 2 |
| **Leung et al. (2018)** | Low | Low | Low | Low | Low | Low | 2 |
| **Moussavi et al. (2019)** | Low | Low | Low | Low | Low | Low | 2 |
| **Stilling, Paxman, et al. (2019)** | Low | Low | Low | Low | Low | Low | 2 |
| **Wilke et al. (2017)** | Low | Low | Low | Low | Low | Low | 2 |

*Note.* Domains: D1 = bias due to randomization, D2 = bias due to deviations from intended intervention, D3 = bias due to missing data, D4 = bias due to outcome measurement, D5 = bias due to selection of reported result. Level of evidence assessment is based on The Oxford 2011 Levels of Evidence (OCEBM Levels of Evidence Working Group, 2011).
