# Supplementary material for: Neuromodulation for Mild Traumatic Brain Injury Rehabilitation: A Systematic Review
Source: Front Hum Neurosci. 2020 Dec 11;14:598208. doi: 10.3389/fnhum.2020.598208 (PMC7759622; doi:10.3389/fnhum.2020.598208)
Supplement: Supplementary file 3 [file Table_3.docx]

Table 3. Risk of bias and level of evidence associated with included non-randomised studies

| Study Authors | D1 | D2 | D3 | D4 | D5 | D6 | Overall Bias | Level of Evidence |
| --- | --- | --- | --- | --- | --- | --- | --- | --- |
| **Ansado, Blunt, Chen, Koski, and Ptito (2019)** | Low | High | N/A | High | High | Low | High | 5 |
| **Fitzgerald et al. (2011)** | N/A | Low | N/A | High | N/A | Low | High | 5 |
| **Huang et al. (2017)** | Low | High | NI | High | N/A | Low | High | 5 |
| **Koski et al. (2015)** | N/A | High | High | High | N/A | High | High | 5 |
| **Leung, Fallah, et al. (2016)** | Low | High | N/A | High | N/A | Low | High | 4 |
| **Paxman, Stilling, Mercier, and Debert (2018)** | N/A | Low | N/A | High | N/A | Low | High | 5 |
| **Stilling, Duszynski, et al. (2019)** | Low | Low | N/A | High | Low | Low | High | 5 |
| **Walker, Norman, and Weber (2002)** | Low | High | Moderate | High | High | High | High | 5 |

*Note.* Domains: D1 = selection bias, D2 = confounding bias, D3 = performance bias, D4 = detection bias, D5 = attrition bias, D6 = selective outcome reporting. Level of evidence assessment is based on The Oxford 2011 Levels of Evidence (OCEBM Levels of Evidence Working Group, 2011), N/A = not applicable, NI = no information. Since there is no recommended way to form a risk of bias judgement for this tool, the authors’ judgement was used.
