# Supplementary material for: Neuromodulation for Mild Traumatic Brain Injury Rehabilitation: A Systematic Review
Source: Front Hum Neurosci. 2020 Dec 11;14:598208. doi: 10.3389/fnhum.2020.598208 (PMC7759622; doi:10.3389/fnhum.2020.598208)
Supplement: Supplementary file 4 [file Table_4.docx]

Table 4

*Summary of Injury Characteristics in Included Studies Evaluating the Efficacy of Neuromodulation in mTBI*

| *Source* | *Injury Description* | | | | | | |  |
| --- | --- | --- | --- | --- | --- | --- | --- | --- |
|  | *Classification* | *Imaging* | *MOI* | *Time since injury* | *LOC* | *PTA* | *GCS* | |
| *Randomised studies* | | | | | | | | |
| G. S. Choi et al. (2018) | mTBI + chronic central pain | Negative | NR | *M*: 15.7 months (*SD*: 7.1). All >6 months post-mTBI | <30 minutes | <24 hours | 13-15 | |
| Leung, Shukla, et al. (2016) | mTBI, PTH | NR | NR | Treatment group *M*: 178 months, *SD*: 176  Control group *M*: 163 months, *SD*: 142 | NR | NR | NR | |
| Leung et al. (2018) | mTBI, PTH | NR | NR | rTMS group *M*:95 months, *SD*: 83  Control group *M*: 99 months, *SD*: 58 | <30 mins | NR | 13-15 | |
| Moussavi et al. (2019) | PPCS | NR | Falls, MVA, Sporting accidents | 4.5 months – 4.8 years | NR | NR | >13 within 10 minutes | |
| Stilling, Paxman, et al. (2019) | PPCS with persistent PTH | NR | MVA (*n*=9), sports (*n*=6), falls (*n*=2), other (*n*=2) | *M*: 32.5 months (>2.5 years), *SD*: 13.9 months, range: 3 months – 5 years | NR | NR | NR | |
| Wilke et al. (2017) | mTBI | NR | Ice hockey (*n*=2), skating (*n*=1), rugby (*n*=1), fall (*n*=2), football (*n*=8), mountain bike (*n*=1), kickboxing (*n*=1), soccer (*n*=1) | 7-54 months | NR | Confusion <24 hours | NR | |
| *Non-randomised studies* | | | | | | | | |
| Ansado et al. (2019) | mTBI with PPCS | NR | NR | 1-28 years | NR | NR | NR | |
| Fitzgerald et al. (2011) | mTBI | NR | MVA | 14 years | <1 hour, fluctuating consciousness for the next 8 hours | NR | NR | |
| Huang et al. (2017) | mTBI + PPCS | NR | MVA (*n*=2), blast (*n*=3), blunt impact (*n*=1) | *M*: 48.2 months (*SD*: 25.2) | <15 minutes | <24 hours | 13-15 | |
| Koski et al. (2015) | mTBI with PCS >3 months | NR | NR | Range: 6 months – 28 years  (66.7% within 3 years of injury, others >8 years post-injury) | NR | NR | NR | |
| Leung, Fallah, et al. (2016) | mTBI-HA | Negative | Fall (*n*=4), MVA (*n*=2) | *M*: 4.4 years (+/-2.2), range: 1.5-7 years | < 1 minute | NR | NR | |
| Paxman et al. (2018) | mTBI | Negative | Pedestrian versus vehicle traffic accident | 5 years | Brief LOC | NR | 15 | |
| Stilling, Duszynski, et al. (2019) | Sports related concussion, concussion | Negative | Sports, MVA | 1-2 years | Nil | Nil | NR | |
| Walker et al. (2002) | MHI | NR | NR | *M*: 12.7 months, *SD*: 18.5, range: 3-70 months | 0-20 minutes | 0-48 hours | NR | |

*Note:* GCS = Glasgow Coma Scale, LOC = loss of consciousness, *M* = mean, MHI = mild head injury, MOI = mechanism of injury, mTBI = mild traumatic brain injury, mTBI-HA = mild traumatic brain injury-related headaches, MVA = motor vehicle accident, NR = not reported, PCS = post concussive symptoms, PPCS = persistent post-concussion syndrome/symptoms, PTA = post-traumatic amnesia, PTH = post-traumatic headache, *SD* = standard deviation.
