# Supplementary material for: Neuromodulation for Mild Traumatic Brain Injury Rehabilitation: A Systematic Review
Source: Front Hum Neurosci. 2020 Dec 11;14:598208. doi: 10.3389/fnhum.2020.598208 (PMC7759622; doi:10.3389/fnhum.2020.598208)
Supplement: Supplementary file 5 [file Table_5.docx]

Table 5

*Summary of Post- Injury Characteristics in Included Studies Evaluating the Efficacy of Neuromodulation in mTBI*

| *Source* | *Post-injury characteristics* | | | | | | | | | | | | |
| --- | --- | --- | --- | --- | --- | --- | --- | --- | --- | --- | --- | --- | --- |
|  | *Duration of symptoms* | *Symptom domains* | | | | | | | | | | *Prior treatment before study* | |
|  |  | *Cognitive* | *Fatigue* | *Sleep disturbance* | *Anxiety/ mood* | *Headache/ migraine* | *Ocular* | *Physical* | *Vestibular* | *Other* |  | |  |
| *Randomised studies* | | | | | | | | | | | | |  |
| G. S. Choi et al. (2018) | >6 months |  |  |  |  |  |  |  |  | Chronic central pain | Pain medication; Pregabalin, Tramadol or Acetaminophen | |  |
| Leung, Shukla, et al. (2016) | Treatment group *M*: 178 months, *SD*: 176  Control group *M*: 163 months, *SD*:142 | Baseline measures showed overall low ratings on reaction time tasks as well as mild dysfunction on omissions, hit reaction time standard error and variability on CCPT II |  |  | Moderate depression ratings (HRSD) | Only included if average chronic daily persistent headache (24/7) was greater than 30/100 intensity on M-VAS and 3/10 on NRS |  | Moderate pain levels |  | Moderate interference with quality of life | Medication management (*n*=21) | |  |
| Leung et al. (2018) | rTMS group *M*: 95 months, *SD*: 83  Control group *M*: 99 months, *SD*: 58 | Baseline measures showed low performance on reaction time tasks, mildly impaired performance on hit reaction time standard error and variability on CCPT II as well as learning and memory deficits on HVLT |  |  | Severe depression ratings (HRSD) and severe to extreme PTSD |  |  |  |  | Moderate interference with quality of life | Medication management^[[1]](#footnote-1)^ (*n*=25) | |  |
| Moussavi et al. (2019) | 4.5 months – 4.8 years |  |  |  | Majority rated as mild depression, 2 rated moderate |  |  |  |  |  | Anti-depressant medication (*n*=4) in PCS group | |  |
| Stilling, Paxman, et al. (2019) | *M*: 32.5 months (>2.5 years), *SD*: 13.9 months, range: 3 months – 5 years |  |  |  |  | PTH: migraine (*n*=20), neck pain (*n*=11), tension (*n*=4) |  |  |  |  | Medications, botox (6-8 weeks prior), | |  |
| Wilke et al. (2017) | 7-24 months | Yes |  |  |  |  |  |  |  |  | NR (CNS altering medications was an exclusion criteria) | |  |
| *Non-randomised studies* | | | | | | | | | | | | |  |
| Ansado et al. (2019) | >6 months | NR | NR | NR | NR | NR | NR | NR | NR | PCSS score of 21+ | NR | |  |
| Fitzgerald et al. (2011) | 13.5 years |  |  | Yes | Yes |  |  | Yes |  |  | Venlafaxine for 8 years, Fluoxetine + Olanzapine for 6 weeks, Desvenlafaxine for 8 weeks (maintained stable dose throughout study). | |  |
| Huang et al. (2017) | *M*:48.2 months, *SD*: 25.2, range: 14-84 months | Yes (*n*=6) | Yes (*n*=4) | Yes (*n*=5) | Yes (*n*=3) | Yes (*n*=5) | Yes (*n*=5) | Yes (*n*=2) | Yes (*n*=4) | Persistent symptoms in minimum 3 categories for inclusion | SSRI (*n*=2) – dosage remained stable throughout study | |  |
| Koski et al. (2015) | 0.5-28 years |  |  |  | 60% elevate depression symptoms, 20% severe |  |  |  |  | PCSS score >21, range: 20-55, *M*: 37.5 | Anti-depressant medication (*n*=4) | |  |
| Leung, Fallah, et al. (2016) | *M*: 4.4 years (+/-2.2), range: 1.5-7 years | *Yes (n*=1) |  |  |  | Yes |  | Neck and back pain (*n*=2) |  |  | Multiple preventative and abortive headache medications and Botox. | |  |
| Paxman et al. (2018) | >5 years |  | Yes |  |  |  |  |  | Yes | Nausea, post-traumatic dizziness of idiopathic origin | Vestibular rehabilitation, vision therapy, venlafaxine, cervical spine rehabilitation (physiotherapy), craniosacral therapy, antidepressant medication | |  |
| Stilling, Duszynski, et al. (2019) | 1-2 years | Yes | Yes | Yes | Yes | Yes (headache) | Yes | Neck pain | Yes | Nausea, dizziness | Patient 1: Craniosacral therapy, physiotherapy, vestibular and vision therapy, medication, nerve blocks, cranial Botox, prism glasses and exercise.  Patient 2: Massage, hyperbaric oxygen chamber therapy, occupational and physical therapy, social work, psychology, productivity consultant, medications, prism glasses, vestibular physiotherapy, vision therapy and exercise. | |  |
| Walker et al. (2002) | *M*: 12.7 months, range 3-70 months | Yes  Memory (72%)  Concentration (44%)  Confusion (8%) | 12% | 12% | Depression (44%)  Anxiety (16%) | 84% | 20% | Neck pain (12%) | Dizziness (12%) |  | NR | |  |

*Note:* CCPT II = Conners’ Continuous Performance Test (Conners, 2004), CNS = central nervous system, EBV = Epstein-Barr virus infection, HRSD = Hamilton Rating Scale for Depression (Hamilton, 1960), HVLT = Hopkins Verbal Learning Test (Brandt, 1991), mTBI = mild traumatic brain injury, M-VAS = mechanical visual analogue scale, NR = not reported, NRS = numerical rating scale, PCS = post-concussion syndrome, PCSS = Post Concussion Symptom Scale (Lovell et al., 2006), PTSD = post-traumatic stress disorder. Note that gaps in the table (i.e., no entry in a column for a particular study), means that the particular symptom was either not reported, or not present in that study.

1. A range of medications types were listed here including anti-depressant, anti-inflammatory and anti-seizure medications, however all were listed for headache management. [↑](#footnote-ref-1)
