# Supplementary material for: Neuromodulation for Mild Traumatic Brain Injury Rehabilitation: A Systematic Review
Source: Front Hum Neurosci. 2020 Dec 11;14:598208. doi: 10.3389/fnhum.2020.598208 (PMC7759622; doi:10.3389/fnhum.2020.598208)
Supplement: Supplementary file 6 [file Table_6.docx]

Table 6A

*Summary of Intervention Protocols in Included Studies Evaluating the Efficacy of Neuromodulation in mTBI*

| *Source* | *Intervention* | | *Outcomes* | | | | |
| --- | --- | --- | --- | --- | --- | --- | --- |
|  | *Intervention protocols* | *Control group intervention* | *Measures used* | *Measurement time points* | *Neurophysiological changes noted* | *Proposed MOA* | *Adverse events* |
| *Randomised studies* | |  |  |  |  |  |  |
| G. S. Choi et al. (2018) | rTMS (10Hz) over primary motor cortex of affected hemisphere.  10 sessions, 5x/week over 2 weeks | Sham rTMS (*n*=6), healthy controls for MRI/DTT STT tract comparison (*n*=30) | DTT, NRS, SF-36 (PCS + MCS scores), HRQOL, MMSE, Brunnstrom Classification and Functional Ambulation Category | Baseline, 5^th^ session, 10^th^ session, 1, 2 and 4 weeks after last intervention session | DTT showed FA and TV values >2SD lower than controls in the spinothalamocortical pathway. Found in all participants at 2.6 months post-mTBI | (i) Modulation of the dysfunctional excitation of the sensory system by the thalamocortical pathway, which triggers analgesic cascades in pain-associated structures  (ii) Restoring cerebral blood flow by increasing blood circulation in low flow areas. | Nil |
| Leung, Shukla, et al. (2016) | rTMS (10Hz) over left motor cortex.  3 sessions over 1 week | Sham rTMS (*n*=12) | Neuropsychological assessments, persistent headache score, debilitating headache score, CCPT II, HRSD, M-PTSD, Global BPI | Baseline, 1-week post treatment and 4 weeks post treatment | NR | Not analysed in this study but thought to be related to alteration of connectivity and excitability of pain modulation/ adaptation-related brain regions by rTMS. | Temporary local tenderness at treatment site (*n*=1), temporary dizziness (*n*=2). |
| Leung et al. (2018) | rTMS (10Hz) over left DLPFC.  4 sessions over 1-2 weeks | Sham rTMS (*n*=15) | Neuropsychological assessments (CCPT II, WAIS-IV, HVLT, Stroop Test), persistent headache score, debilitating headache score, BPI, CAPS | Baseline, 1-week post treatment and 4 weeks post treatment | NR | It is thought that the DLPFC stimulation incudes analgesic effects through top-down inhibition via the descending PFC-midbrain-thalamic-cingulate pathway as well as its interactions with the motor cortex.  Stimulation of the motor cortex is also thought to alter the connectivity and excitability of pain modulation/ adaptation-related brain regions. | Temporary elevation of perseverations score. |
| Moussavi et al. (2019) | rTMS (20Hz) over left DLPFC.  13 sessions over 3 weeks | Sham rTMS (*n*=9) | RPCSQ, MADRS, EVestG | Baseline, end of treatment, 30 and 60 days | NR | It is thought that rTMS modifies the rate of recovery rather than directly affecting symptoms, by modulating network neuroplasticity on a long-term basis.  Hence rTMS is thought to promote brain recovery. | Mild headache (*n*=5), headache worsening after 1 day of treatment (*n*=1) |
| Stilling, Paxman, et al. (2019) | rTMS (10Hz) over left DLPFC.  10 sessions, 5x/week over 2 weeks | Sham rTMS | Headache frequency, headache severity (NRS), daily headache diary, clinical questionnaires; HIT-6, MoCA, PHQ-9, GAD-7, PCL-5, RPCSQ, QOLIBRI, BCPSI | Baseline, end of treatment (day 14), 1, 3- and 6-months post rTMS |  | Potential role of DLPFC in PTH via pain modulation. | Mild headache aggravation (4.23%), scalp discomfort (0.96%), dizziness (0.3%) and toothache (0.68%).  One control participant experienced altered facial sensation contralateral to stimulation. |
| Wilke et al. (2017) | atDCS (1mA) over left primary motor cortex for 20 minutes.  1 session (7 days before/after sham) | atDCS Sham, current was turned off after 30 seconds.  1 session | MRS, TMS, PSPS, cognitive testing, RDS, MMSE, BDI | Before and after atDCS, before and after sham |  |  | Nil |
| *Non-randomised studies* | |  |  |  |  |  |  |
|  |  |  |  |  |  |  |  |
| Ansado et al. (2019) | rTMS (10Hz) over left DLPFC.  20 sessions, 5x/week over 4 weeks | N/A | fMRI + dMRI of CC during verbal + visual wm task, PCSS | Before and after rTMS | Microstructural CC changes leading to alterations in wm activation patterns | Restoration of inter-hemispheric balance of the wm network. | Worsening symptoms after a few weeks (*n*=2) |
| Fitzgerald et al. (2011) | rTMS 10Hz to left DLPFC and 1Hz to right DLPFC.  20 sessions, 5x/week over 4 weeks | N/A | MADRS, MRI, DTI, neuropsychology assessment, Sheehan Disability Scale | Baseline, 2 weeks, 4 weeks (end of treatment) | NR | Modulation of dorsal frontal-subcortical limbic connections | Nil |
| Huang et al. (2017) | Neurofeedback (IASIS) with LIP-tES and MEG monitoring, 12 sessions, 2x/week over 6 weeks.  5 EEG electrodes used with 3 standard placement protocols | N/A | RPCSQ, resting state MEG | Baseline, after intervention (6 weeks) | Excessive slow wave (delta 1-4Hz) EEG activity | Possibly via the phenomenon of stochastic resonance – boosting a naturally occurring oscillation signal within the brain using electrical current. This enhanced endogenous slow wave activity is thought to encourage healing within the brain. | Nil |
| Koski et al. (2015) | rTMS (10Hz) over left DLPFC.  20 sessions, 5x/week over 4 weeks | N/A | MRI, fMRI, PCSS-22, self-report CSQ, neuropsychological testing, | Baseline, within 2 weeks post-rTMS and 3 months after last rTMS session | Altered DLPFC activation during working memory tasks. | Enhancing functional activation of DLPFC | Worsening symptoms (*n*=2), side effects: headaches (*n*=3), vertigo (*n*=1), sleep disturbance (*n*=3), sensitivity to stimulation intensity for first week (*n*=9) |
| Leung, Fallah, et al. (2016) | rTMS (10Hz) over left DLPFC and left motor cortex.  4 sessions +  monthly maintenance sessions (*n*=4) | N/A | Headache frequency, duration and intensity | Before and after rTMS | NR | NR | Nil |
| Paxman et al. (2018) | rTMS (10Hz) over left DLPFC.  10 sessions over 2 weeks | N/A | Brain MRI and MRI angiogram, cervical spine MRI, neuropsychological assessment, vestibular evoked myogenic potential, computerised dynamic posturography, VNG, physical examination, vision assessment, blood analysis, heavy metal screen, DHI, QOLIBRI | Baseline, end of treatment, 1- and 3-months post treatment | NR | rTMS is thought to decrease elevated cortical excitation, induce long-term plasticity at the synaptic level. Specifically, it is thought to modulate postural control and the perception of motion through vestibulo-cortical projections. | Temporary fatigue. |
| Stilling, Duszynski, et al. (2019) | rTMS (10Hz) over left DLPFC.  10 sessions over 2 weeks | 2 sex-matched controls for imaging comparison. | 8-week headache diary, clinical questionnaires, fNIRS, physical examination, CT head, neuroendocrine testing, HIT-6, MoCA, PHQ-9, GAD-7, PCL-5, RPCSQ, QOLIBRI, BCPSI | Baseline, after rTMS (day 14), 1 month after rTMS | One participant displayed an abnormal haemodynamic activation in the DLPFC during a wm task | rTMS alters blood flow and microstructure at the DLPFC which improves function and is associated with improved clinical symptoms. | Nil |
| Walker et al. (2002) | 2-channel qEEG guided NFT targeting coherence.  5 session sets, sessions continued until symptoms reduced or until 40 sessions.  (*M*: 19 sessions, *SD*: 9.7, range: 5-41) | N/A | QEEG (eyes closed), self-report global improvement score (GIS; percentage change from 0-100%), clinical history | Baseline, end of each 5-session set. | NR | NR | Nil |

*Note:* atDCS = anodal transcranial direct current stimulation, BCPSI = British Columbia Post-Concussion Symptom Inventory (Iverson, Zasler, & Lange, 2007), BDI = Beck Depression Inventory (Hautzinger, Keller, & Kühner, 2006), BPI = Brief Pain Inventory (Cleeland & Ryan, 1994), Brunnstrom Classification and Functional Ambulation Category (Cho et al., 2012), CAPS = Clinical Administered Post-Traumatic Stress Disorder Scale (Bormann, Oman, Walter, & Johnson, 2014), CC = corpus callosum, CCPT II = Conner’s Continuous Performance Test II (Conners, 2004), CSQ = Cognitive Symptoms Questionnaire (Koski et al., 2015), DHI = Dizziness Handicap Inventory (Tamber, Wilhelmsen, & Strand, 2009), DLPFC = dorsolateral prefrontal cortex, dMRI = diffusion MRI, DTI = diffusion tensor imaging, DTT = diffusion tensor tractography, EVestG = electrovestibulography, FA = fractional anisotropy, fNIRS = functional near-infrared spectroscopy, GAD-7 = Generalized Anxiety Disorder Scale -7 (Spitzer, Kroenke, Williams, & Lowe, 2006), HIT-6 = Headache Impact Test -6 (Kosinski et al., 2003), HRQOL = Health Related Quality of Life, HRSD = Hamilton Rating Scale for Depression (Hamilton, 1960), HVLT = Hopkins Verbal Learning Test (Brandt, 1991), IASIS = microcurrent neurofeedback program (J & J Engineering), LIP-tES = low intensity pulses using transcranial electrical stimulation, MADRS = Montgomery-Asberg Depression Rating Scale (Montgomery & Asberg, 1979), MCID = minimal clinically important difference, MEG = magnetoencephalography, MMSE = Mini Mental State Examination (Folstein, Folstein, & McHugh, 1975), MOA = mechanism of action, MoCA = Montreal Cognitive Assessment (Nasreddine et al., 2005), M-PTSD = Mississippi scale for Post-Traumatic Stress Disorder (Keane, Caddell, & Taylor, 1988), MRI = magnetic resonance imaging, MRS = magnetic resonance spectroscopy, mTBI = mild traumatic brain injury, N/A = not applicable, NFT = neurofeedback therapy, NR = not reported, NRS = numerical rating scale, PCL-5 = Post Traumatic Stress Disorder Checklist for DSM-5 (Blevins, Weathers, Davis, Witte, & Domino, 2015), PCSS = Post Concussion Symptom Scale (Lovell et al., 2006), PFC = prefrontal cortex, PHQ-9 = Patient Health Questionnaire -9 (Kroenke, Spitzer, & Williams, 2001), QOLIBRI = Quality of Life After Brain Injury Questionnaire (von Steinbüchel et al., 2010), RPCSQ = Rivermead Post-Concussion Symptom Questionnaire (King, Crawford, Wenden, Moss, & Wade, 1995), rTMS = repetitive transcranial magnetic stimulation, SF-36 = Short Form 36 Health Survey (Ware, Dewey, & Kosinski, 2001), SF-36 MCS = mental component score, SF-36 PCS = physical component score, Sheehan Disability Scale (Sheehan, Harnett-Sheehan, & Raj, 1996), SPC = superior parietal cortex, STT = spinothalamocortical tract, TMS = transcranial magnetic stimulation, TV = tract volume, VLPFC = ventrolateral prefrontal cortex, VNG = videonystagmography, WAIS-IV – Wechsler Adult Intelligence Scale 4^th^ Edition (Wechsler, Pearson Education, & PsychCorp, 2009), wm = working memory. *Underlined measures are self-report measures used in a non-blinded study.

Table 6B

*Summary of Outcomes in Included Studies Evaluating the Efficacy of Neuromodulation in mTBI*

| *Source* | *Outcomes* | *Intervention group* | | | | | | *Control Group* | | | | |
| --- | --- | --- | --- | --- | --- | --- | --- | --- | --- | --- | --- | --- |
|  |  | *Modality* | *Statistical method* | *Mean* | *SD* | *Sig.* | *Effect size* | *Modality* | *Mean* | *SD* | *Sig.* | *Effect size* |
| Randomised studies | | | | | | | | | | | | |
| G. S. Choi et al. (2018) | (1) Pain intensity was lower in rTMS versus control group at post intervention measures (time 1-5) | rTMS | GLM analysis with Bonferroni correction | - | - | *p* < 0.001 | - | Sham rTMS | N/A | N/A | N/A | N/A |
|  | (2) SF-36 physical component score had a higher increase in rTMS versus control group at post intervention measures (time 2-5) |  |  | - | - | *p* < 0.001 | - |  | N/A | N/A | N/A | N/A |
| Leung, Shukla, et al. (2016) | (1) Treatment group had a significantly greater reduction in persistent headache intensity compared to controls at 1- and 4-week post-treatment. | rTMS | 2-factor, RM ANOVA, ANCOVA | 56.3% | 48.2 | *p* = 0.041 | - | Sham rTMS | 15.4% | 43.6 | *p* = 0.041 | - |
|  | (2) A significantly higher percentage of the treatment group participants had at least 50% reduction in headache intensity compared to controls at 1-week post treatment. |  |  | 58.3% had 50%+ reduction | - | *p* = 0.035 | - |  | 16.6% had 50%+ reduction | - | *p* = 0.035 | - |
|  | (3) Overall score of debilitating headaches was significantly reduced at 4-weeks post-treatment in treatment group. |  |  | Pre: 31.7,  Post: 4.2 | Pre: 35.4,  Post: 3.2 | *p* = 0.017 | - |  | Post: 16 | - | - | - |
|  | (4) No changes were seen on a sustained attention measure at 4-weeks post treatment. |  | RM ANOVA | - | - | - | - |  | - | - | - | - |
| Leung et al. (2018) | (1) Treatment group showed significant reduction in headache intensity at 1 week and 4 weeks post treatment compared to controls. | rTMS | 2-factor RM ANOVA | 1 week: 25.3%  Pre: 4.9  Post: 3.5  4 weeks: 23% (SD: 17.7),  Pre: 4.9  Post: 3.6 | 1 week: 16.8%  Pre: 1.7  Post: 2.0  4 weeks: 17.7%  Pre: 1.7  Post: 2.0 | 1 week: *p* < 0.0001  4 weeks:  *p* < 0.01 | - | Sham rTMS | 1 week:  <1%  4 weeks:  2.3%,  Pre: 4.8  Post: 4.7 | 1 week: 11.7%  4 weeks: Pre:1.5  Post: 1.7 | - | - |
|  | (2) There was a significant reduction in prevalence of persistent headaches in both the treatment and control groups at 1- and 4-weeks post treatment. |  | ANOVA | 1 week = 50% no headaches,  4 weeks = 57% no headaches | - | *p* = 0.009 | - |  | 1 week = 7% no headaches,  4 weeks = 20% no headaches | - | *p* = 0.009 | - |
|  | (3) Treatment group had a significant reduction in depression scores at 1-week post treatment however this was no longer significant at 4- weeks post treatment.  The control group did not show a significant reduction in depression symptoms at 1- and 4-weeks post treatment. |  | ANOVA | Very severe to moderate/lower severe, 22.3 to 19.0 | Pre: 6.4  Post: 5 | *p* = 0.033 | - |  | Very severe, 25.3 to 24.6 | Pre: 8.4  Post: 5 | *p* = 0.033 | - |
| Moussavi et al. (2019) | (1) Individuals <12 months post injury showed significant reduction in PCS at 30 (post 1) and 60 days (post 2) follow up compared to controls and those with greater time post-injury. | rTMS | Double multivariate analysis of RM ANOVA | RPQ3:  Pre: 10.3  Post -5  Post 2: -6.17  RPQ13:  Pre: 41.5  Post 1: -21.83  Post 2: -21.17 | RPQ3:  Pre:1.7  Post 1: 3.12 Post 2: 1.76  RPQ13:  Pre: 6.9  Post 1: 6.6  Post 2: 5.3 | *p* = 0.001 | RPQ3: Cohen’s d  Post 1: 0.00  Post 2: -0.74  RPQ13:  Cohen’s d  Post 1: -2.35  Post 2: -1.42 | Sham rTMS | RPQ3:  Pre: 8.9  Post 1: -5  Post 2: -4.63  RPQ13:  Pre: 34.6  Post 1: -2.16  Post 2: -5.38 | RPQ3:  Pre: 3.4  Post 1: 1.73  Post 2: 2.29  RPQ13:  Pre: 5.7  Post 1: 9.83  Post 2: 13.65 | *p* = 0.001 | See treatment group section for effect sizes |
|  | (2) EVestG improvement was seen in the treatment group and was significantly different to controls at the 60 days follow up in the <12 months since injury group. |  |  | Pre: 19.8  Post: 22.05 | Pre: 2.1  Post: 8.78 | - | Cohen’s d = 1.27 |  | Pre: 20.0  Post: 7.81 | Pre: 9.3  Post: 13.24 | - | See treatment group section for effect size |
|  | (3) Depression ratings improved significantly for both the treatment and control groups in the <12 months since injury group, but not in the longer duration since injury group. |  |  | Pre:19.0  Post 1: 6.0  Post 2: 5.0 | Pre: 5.7  Post 1: 2  Post 2: 1 | - | Cohen’s d  Post 1: 0.47 Post 2: -1.01 |  | Pre: 20.3 Post 1: 12.3  Post 2: 17.0 | Pre: 12.9  Post 1: 12.0 Post 2: 12.7 | - | See treatment group section for effect size |
| Stilling, Paxman, et al. (2019) | (1) Average headache severity was reduced in the treatment and control group, but these were below the MCID. | rTMS | 2-way mixed ANOVA | Pre: 4.42  Post: 4.21 | Pre: 1.2 Post: 1.64 | *p* = 0.030 | Hedges’ g = 0.182 | Sham rTMS | Pre: 5.09 Post: 4.68 | Pre: 0.62  Post: 1.17 | - | - |
|  | (2) Average headache frequency reduced in the treatment and control groups. |  | Non-parametric | Pre: 35.4  Post: 30.2 | Pre: 8.4  Post: 12.6 | - | Hedges’ g = -0.267 |  | Pre: 28.5  Post: 25.2 | Pre: 11.9  Post: 14.6 | - | Hedges’ g = -0.267 |
|  | (3) A larger proportion of the treatment group returned to work compared to the control group. |  | Descriptive analysis | 60% of participants | - | *p* = 0.027 | - |  | 10% of participants | - | *p* = 0.027 | - |
|  | (4) Headache functional impact (HIT-6) improved immediately post-treatment (post 1) and at 1 month follow up (post 2) in the treatment group. However, the MCID was not met. |  | 2-way mixed ANOVA | Post 1: 5.5 points  Post 2: 5.6 points | - | *p* = 0.002 | Hedges’ g = -0.44 |  | Post 1: -  Post 2: 3.7 points | - | *p* = 0.046 | Hedges’ g = -0.44 |
|  | (5) Post concussion symptoms (RPCSQ) decreased at 1 month follow up in the treatment and control groups. |  | 2-way mixed ANOVA | RPSQ-3: -1.6 | - | *p* = 0.031 | Hedges’ g = -0.46 |  | RPSQ-3:  -2 | - | *p* = 0.006 | Hedges’ g = -0.46 |
|  | (6) Depression (PHQ-9) and quality of life (QOLIBRI) ratings decreased significantly in the treatment group at 1 month follow up, however the amount of change did not meet the MCID. |  |  | PHQ-9: -4.3  QOLIBRI: -5.6 | 3.7  - | *p* = 0.020  - | Hedges’ g = 0.31 |  | PHQ-9: -0.7  QOLIBRI: -1.9 | 4.7  - | *p* = 0.027  - | Hedges’ g = 0.31 |
|  | (7) Some participants stopped Botox treatment in the treatment (*n*=1) and the sham (*n*=2) group. |  | - | - | - | - | - |  | - | - | - | - |
|  | (8) Some participants decreased or stopped medication in the treatment group (*n*=3). |  | - | - | - | - | - |  | - | - | - | - |
| Wilke et al. (2017) | (1) atDCS did not influence GABA concentration or GABA_B_ receptor activity in the primary motor cortex. | atDCS | 2x2 RM ANOVA | - | - | *p* = 0.88 (time)  *p* = 0.41 (condition) | - | Sham atDCS | - | - | - | - |
| Non-randomised studies | | | | | | | | | | | | |
| Ansado et al. (2019) | (1) Wm network activation changed from unilateral to bilateral, | rTMS | 2-way RM ANOVA with Bonferroni correction | - | - | - | - | N/A | N/A | N/A | N/A | N/A |
|  | (2) Effect of the anterior fibres of the CC on the right wm network changed from inhibition to activation |  | 2-way RM ANOVA | Before = CC and right frontoparietal pathway (r = -0.29), right VLPFC (r = -0.28), right SPC (r = -0.43), right DLPFC (r = 0.27).  After = CC and R frontoparietal pathway (r = 0.39), right DLPFC (r = 0.33), right VLPFC (r = 0.28, right RPFC (r = 0.37, right SPC (r = 0.31). | - | - | - |  | N/A | N/A | N/A | N/A |
|  | (3) Effect of posterior fibres of CC on the right wm network changed from activation to inhibition. (which CC division/s not specified) |  | - | - | - | - | - |  | N/A | N/A | N/A | N/A |
| Fitzgerald et al. (2011) | Sheehan Disability Scale changed from marked-extreme at baseline, to mild-moderate after rTMS. | rTMS | - | 50% | - | - | - | N/A | N/A | N/A | N/A | N/A |
|  | Improvements seen in some cognitive tasks (verbal fluency and information processing speed), slight decrease in attention task performance. |  | - | Only raw scores or percentiles provided, no MCID calculated | - | - | - |  | N/A | N/A | N/A | N/A |
| Huang et al. (2017) | (1) Reduced RPCSQ total score | NFT | Paired group t-test | 52.6%, *t* = 5.80 | 26.4 | *p* < 0.01 | Cohen’s d = 2.37 | N/A | N/A | N/A | N/A | N/A |
|  | (2) Sleep disturbance sub-score significantly reduced |  |  | *t* = 3.00 |  | *p* < 0.05 | Cohen’s d = 1.22 |  | N/A | N/A | N/A | N/A |
|  | (3) Reduced abnormal MEG slow wave z-score |  |  | 53.6%, *t* = 4.28 | 24.6 | *p* < 0.01 | Cohen’s d = 1.75 |  | N/A | N/A | N/A | N/A |
| Koski et al. (2015) | (1) Reduced average PCS scores reduced – greater change in older participants. | rTMS | - | 14.6 | 16.1 | *p* = 0.009 | Effect size = 0.91 | N/A | N/A | N/A | N/A | N/A |
|  | (2) Increase in activation of DLPFC during task-related fMRI |  | Whole brain subtraction analysis | - | - | - | - |  | N/A | N/A | N/A | N/A |
|  | (3) No significant changes noted on Cognitive Symptoms Questionnaire or neuropsychological testing |  |  | -155.7 (mean change) | 320.1 | - | - |  | N/A | N/A | N/A | N/A |
| Leung, Fallah, et al. (2016) | (1) Headache intensity was reduced | rTMS | - | 53.05% | 19.9 | - | - | N/A | N/A | N/A | N/A | N/A |
|  | (2) Headache frequency was reduced, frequency = 0 (*n*=2) |  | - | 78.97% | 19.88 | - | - |  | N/A | N/A | N/A | N/A |
|  | (3) Headache duration was reduced for those who did not have complete cessation/ zero frequency (n=4). Headache intensity was also reduced. |  | - | 50% (duration)  31.7% (frequency) | - | - | - |  | N/A | N/A | N/A | N/A |
|  | (4) Headache abortive medication was completely stopped by 3 participants and the remaining 3 participants reduced their medications. |  | - | - | - | - | - |  | N/A | N/A | N/A | N/A |
| Paxman et al. (2018) | (1) Dizziness frequency and severity was reduced at 3 months follow up | rTMS | MCID where applicable | >50% reduction  Pre: 40/100  Post: 26/100  (MCID = 11) | - | - | - | N/A | N/A | N/A | N/A | N/A |
|  | (2) Dizziness severity reduced at 1 month (post 1) and 3 months (post 2) follow up. |  |  | Pre: 6.14/10  Post 1: 5/10  Post 2: 3/10 | - | - | - |  | N/A | N/A | N/A | N/A |
|  | (3) Dizziness frequency reduced 1 month (post 1), and 3 months (post 2) follow up. |  |  | 58.5 episodes/week  Post 1: 48 episodes Post 2: 21 episodes | - | - | - |  | N/A | N/A | N/A | N/A |
|  | (4) Impact on quality of life improved (DHI score reduced), no longer meeting the cut-off for disability (29/100) at the 3 months follow up. |  |  | DHI score reduced by 14 points | - | - | - |  | N/A | N/A | N/A | N/A |
|  | (5) Quality of life on QOLIBRI showed slight improvement at 1 month (post 1) and 3 months (post 2) follow up. |  |  | Pre: 88/100  Post 1: 93/100  Post 2: 95/100 | - | - | - |  | N/A | N/A | N/A | N/A |
| Stilling, Duszynski, et al. (2019) | (1) Abnormal fNIRS haemodynamic response at DLPFC during wm task was normalised at 1-month post-treatment and this was associated with improved clinical outcomes (*n*=1). | rTMS | GLM | - | - | - | - | 2 sex-matched controls for imaging comparison. | N/A | N/A | N/A | N/A |
|  | (2) Clinical scores for patient 1 showed minor improvements which did not meet MCID.  Clinical scores for patient 2 show improvements reaching MCID in headache frequency, HIT-6 (functional impact), PHQ-9 (depression) and GAD-7.  Both participants showed overall improvements in QOLIBRI, RPSQ-13 and BCPSI. |  | MCID where applicable | Pt 1: QOLIBRI:  53 (severe) to  66 (moderate)  RPSQ-13: 25 to 22  BCPSI: 65 to 47  Pt 2:  HIT-6: 68 (severe) to 60 (severe).  PHQ-9: 25 (severe) to 8 (mild)  GAD-7: 18 (severe) to 10 (moderate)  QOLIBRI: 6 (severe) to 31 (severe)  RPSQ-13: 48 to 22  BCPSI: 106 to 59 | - | - | - | N/A | N/A | N/A | N/A | N/A |
|  | (3) 1 participant returned to work and 1 maintained part time work after treatment. |  | - | - | - | - | - | N/A | N/A | N/A | N/A | N/A |
| Walker et al. (2002) | (1) Improvement of >50% in global improvement score was seen in 88% of participants. | NFT | Descriptive statistics | 72.7% | 27.6 | - | - | N/A | N/A | N/A | N/A | N/A |
|  | (2) All participants who were employed prior to injury returned to work after NFT. |  |  | - | - | - | - |  | N/A | N/A | N/A | N/A |
|  | (3) Outcomes were not correlated with age or time since injury. |  | Correlation | - | - | - | - |  | N/A | N/A | N/A | N/A |

*Note: ‘*-‘ = information not available, ANOVA = analysis of variance, ANCOVA = analysis of covariance, atDCS = anodal transcranial direct current stimulation, BCPSI = British Columbia Post-Concussion Symptom Inventory (Iverson, Zasler, & Lange, 2007), CC = corpus callosum, DHI = Dizziness Handicap Inventory (Tamber, Wilhelmsen, & Strand, 2009), DLPFC = dorsolateral prefrontal cortex, EVestG = electrovestibulography, GAD-7 = Generalized Anxiety Disorder Scale -7 (Spitzer, Kroenke, Williams, & Lowe, 2006), GLM = generalised linear model, HIT-6 = Headache Impact Test -6 (Kosinski et al., 2003), MCID = minimal clinically important difference, MEG = magnetoencephalography, N/A = not applicable, NFT = neurofeedback therapy, PFC = prefrontal cortex, PHQ-9 = Patient Health Questionnaire -9 (Kroenke, Spitzer, & Williams, 2001), PSPS = Pittsburgh Steelers Post-Concussion Scale (Maroon et al., 2000), QOLIBRI = Quality of Life After Brain Injury Questionnaire (von Steinbüchel et al., 2010), RDS = Reliable Digit Span (Meyers & Volbrecht, 1998), RM ANOVA = repeated measures analysis of variance, RPCSQ = Rivermead Post-Concussion Symptom Questionnaire (King, Crawford, Wenden, Moss, & Wade, 1995), rTMS = repetitive transcranial magnetic stimulation, SF-36 = Short Form 36 Health Survey (Ware, Dewey, & Kosinski, 2001), Sheehan Disability Scale (Sheehan, Harnett-Sheehan, & Raj, 1996), SPC = superior parietal cortex, VLPFC = ventrolateral prefrontal cortex, VNG = videonystagmography, wm = working memory. *Yellow cells relate to pain or headaches.
